# Nucleotide Excision Repair, Mismatch Repair, and R-Loops Modulate Convergent Transcription-Induced Cell Death and Repeat Instability

**DOI:** 10.1371/journal.pone.0046807

**Published:** 2012-10-03

**Authors:** Yunfu Lin, John H. Wilson

**Affiliations:** Verna and Marrs McLean Department of Biochemistry and Molecular Biology, Baylor College of Medicine, Houston, Texas,United States of America; University of Florida, United States of America

## Abstract

Expansion of CAG•CTG tracts located in specific genes is responsible for 13 human neurodegenerative disorders, the pathogenic mechanisms of which are not yet well defined. These disease genes are ubiquitously expressed in human tissues, and transcription has been identified as one of the major pathways destabilizing the repeats. Transcription-induced repeat instability depends on transcription-coupled nucleotide excision repair (TC-NER), the mismatch repair (MMR) recognition component MSH2/MSH3, and RNA/DNA hybrids (R-loops). Recently, we reported that simultaneous sense and antisense transcription–convergent transcription–through a CAG repeat not only promotes repeat instability, but also induces a cell stress response, which arrests the cell cycle and eventually leads to massive cell death via apoptosis. Here, we use siRNA knockdowns to investigate whether NER, MMR, and R-loops also modulate convergent-transcription-induced cell death and repeat instability. We find that siRNA-mediated depletion of TC-NER components increases convergent transcription-induced cell death, as does the simultaneous depletion of RNase H1 and RNase H2A. In contrast, depletion of MSH2 decreases cell death. These results identify TC-NER, MMR recognition, and R-loops as modulators of convergent transcription-induced cell death and shed light on the molecular mechanism involved. We also find that the TC-NER pathway, MSH2, and R-loops modulate convergent transcription-induced repeat instability. These observations link the mechanisms of convergent transcription-induced repeat instability and convergent transcription-induced cell death, suggesting that a common structure may trigger both outcomes.

## Introduction

Tandem repetitive sequences, which are the major constituents of the telomeres and centromeres of chromosomes, are distributed throughout the human genome [1]. Expansions of CAG•CTG tracts in any one of several specific human genes lead to disorders, typically characterized by neurodegeneration, due to loss or death of neurons in disease-specific regions of the brain. So far, thirteen trinucleotide (TNR) disorders have been found to be caused by expansion of a CAG•CTG tract, including Huntington disease (HD), HD-like 2 (HDL2), myotonic dystrophy type 1 (DM1), spinal and bulbar muscular atrophy (SBMA), dentatorubral-pallidoluysian atrophy (DRPLA), and the spinocerebellar ataxias SCA1, SCA2, SCA3, SCA6, SCA7, SCA8, SCA12, and SCA17 [2], [3], [4]. The molecular basis for these CAG repeat diseases (CAG diseases, hereafter) is the expansion of a repeat tract beyond a disease-specific threshold number of repeat units. For reasons that are not entirely clear, long CAG repeat tracts become unstable, with a strong bias toward expansion, both in germline and somatic cells [5]. Expansion in the germline leads to longer repeats in the progeny, along with increased disease severity and earlier age of onset of disease symptoms, while expansion in somatic cells, especially in neurons, accelerates disease progression [3], [4], [6], [7].

One critical topic for understanding and treating CAG diseases is the mechanism of CAG repeat expansion during germline transmission and in somatic cells. Using bacteria, yeast, flies, mammalian cells, and mouse model systems, previous studies have shown that repeat instability can occur in connection with virtually any DNA metabolic pathway, including DNA replication, DNA repair, recombination, and transcription [6], [8], [9], [10], [11], [12]. These processes may vary in their relative importance to repeat instability in different cell types in humans [3], [5], [13]. For example, DNA replication is expected to be a more important contributor to repeat instability in proliferating germ cells than in terminally differentiated neurons [12]. Several genetic observations in mouse models support the idea of multiple, tissue-specific mechanisms for repeat instability: deletion of one copy of the *Dnmt1* (DNA methyltransferase 1) gene increases instability in the male and female germlines, but not in somatic cells [14]; nulls for a component of base excision repair, Ogg1 (8-oxoguanine glycosylase), reduce instability in somatic tissues, but do not affect the germline [15], [16]; and knockout of the *Xpa* gene–which encodes a key component of nucleotide excision repair (NER)–virtually eliminates repeat instability in several specific brain regions, but does not affect instability in liver, kidney, or either germline [17]. These studies indicate that distinct pathways are involved in driving repeat instability in specific tissues.

Studies in human cells and Drosophila initially showed that transcription, in association with DNA repair, promotes CAG instability in eukaryotic cells [18], [19]. It is thought that transcription, by transiently exposing single DNA strands, allows long CAG repeat tracts to form abnormal secondary structures such as hairpins and slipped-strand DNA duplexes, which then engage DNA repair processes [20], [21]. Detailed studies in human cells have shown that transcription-coupled nucleotide excision repair (TC-NER), which specifically removes DNA lesions that block RNA polymerase II (RNAPII), plays a critical role in destabilizing repeats [22], [23]. A recent biochemical study in cell-free extracts has provided support for our genetic observations, by showing that repeat hairpins on either the transcribed or non-transcribed strands can arrest RNAPII [24]. Interestingly, hairpins alone do not arrest pure T7 RNAP, but require additional components in the nuclear extract [24]. The mismatch repair (MMR) recognition complex MSH2/MSH3 is a strong candidate for this activity because it binds to CAG and CTG hairpins [25], [26], plays a crucial role in CAG repeat instability in mice [27], [28], [29], [30], and promotes transcription-induced repeat instability in human cells [18], [31]. In addition, we have identified other modulators of transcription-dependent repeat instability in human cells that may also contribute, including RNA/DNA hybrids (R-loops) [32], the proteasome machinery [23], and the single-strand break repair (SSBR) pathway [33]. These studies indicate that the CAG repeat instability triggered by transcription results from a complex molecular process.

To add to this complexity, two recent papers reported that simultaneous sense and antisense transcription–convergent transcription–through a CAG tract destabilizes the repeats in human cells [34], [35], with larger effects than the sum of sense and antisense transcription alone [35]. The mechanism for convergent transcription-induced repeat instability has not been characterized, but it could plausibly involve the same DNA processes as sense transcription. Convergent transcription, however, not only promotes repeat instability, it also triggers cell-cycle arrest and massive apoptosis-dependent cell death via a DNA damage-like response involving the ATR pathway and its downstream targets such as cell-cycle checkpoint kinase 1 (CHK1) and p53 [35]. In this study, we used siRNA knockdown to define the roles of DNA repair components in convergent transcription-induced repeat instability and cell death. We find that depletion of MSH2 decreases repeat instability and cell death, while depletion of RNase H increases both instability and death. In contrast, depletion of XPA decreases instability, but increases cell death. The possible roles of these proteins in convergent transcription-induced cell death and repeat instability are discussed.

## Materials and Methods

### Cell Lines and Cell Culture

**Figure 1:**
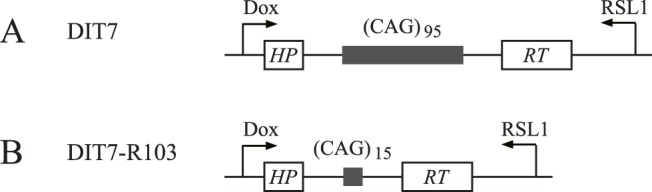
Structure of the HPRT minigenes in DIT7 and DIT7-R103 cells. DIT7 cells carry a CAG_95_ repeat tract and DIT7-R103 cells, which were derived from DIT7 cells by contraction of the repeat, carry a CAG_15_ repeat tract. In both cell lines, the CAG tract is centered in the 2.1-kb intron in the single, randomly integrated *HPRT* minigene. The CAG repeat is about 1.6 kb downstream of the sense promoter and about 2.5 kb upstream of the antisense promoter.

The construction of DIT7 cells was described previously [35]. Briefly, RS11 cells express the rtTA protein, a fusion of the reverse tetracycline repressor protein and the HSV VP16 transcription activation domain, which drives expression from the pTRE-CMV^mini^ promoter in the presence of the inducer, doxycycline. RS11 cells also contain genes for RheoReceptor-1 and RheoActivator, which drive expression from the pNERB-X1 promoter in the presence of the inducer, RSL1. DIT7 cells were derived from RS11 cells by integration of a single copy of an *HPRT* minigene carrying a CAG_95_ tract in its intron, with sense and antisense transcription controlled by the promoters pTRE-CMV^mini^ and pNERB-X1, respectively (Figure 1). Sense transcription of the *HPRT* minigene in DIT7 cells is induced 22-fold with doxycycline, and antisense transcription is induced 16-fold with RSL1 [35]. DIT7-R103 cells were derived from DIT7 by contraction of the CAG repeat tract from 95 to 15 units [35]. Both DIT7 and DIT7-R103 cells were grown at 37°C with 5% CO_2_ in DMEM/F-12 medium supplemented with 10% fetal bovine serum and 1% MEM nonessential amino acids.

### Induction of Transcription

Cells were grown and maintained in the absence of transcription inducers. For all experiments, transcription was induced by addition of inducers on day 0. Sense transcription of the *HPRT* minigene was induced by addition of doxycycline at a final concentration of 0.2 µg/mL. Because the half-life of doxycycline in medium is about 24 hours, 0.1 µg/mL of doxycycline was added into the medium each day until the treatment was completed. Antisense transcription was induced by addition of RSL1 at a final concentration of 500 nM. No additional RSL1 was required.

### siRNA Treatment

About 100,000 cells were plated in each well of a 6-well plate on day −3. On day −2, cells were transfected with individual siRNAs at a final concentration of 200 nM, using oligofectamine (Invitrogen). Treatments with 200 nM vimentin siRNA served as controls. Treatment with vimentin siRNA does not affect the cells; it does not alter the percentage of DIT7 and DIT7-R103 cells that die when convergent transcription is induced. On day 0, cells were again transfected with siRNA, and cultures were then grown in the presence or absence of doxycycline plus RSL1. The efficiency of knockdown of target genes was determined on day +1 for individual siRNAs using real-time RT-PCR, as described previously [23], [32]. All siRNAs used in this study lowered the efficiency of target gene expression by at least 70%. Distinct siRNAs that are targeted to different regions of the same gene are labeled −1 and −2; for example, XPA-1 and XPA-2 indicate two different siRNAs against the XPA gene. The sequences of these siRNAs and RT-PCR primers are identical to those used previously [23], [32].

### Measurements of Dead Cells and Viable Cells

We define adherent cells (attached to the plate) as viable cells, and nonadherent cells (present in the medium) as dead cells [35]. Previously, we showed that fewer than 4% of adherent cells incorporated propidium iodide, indicating that greater than 96% of adherent cells are viable [35]. By contrast, more than 99% of nonadherent cells stained with propidium iodide. The small contamination of nonadherent cells by live cells (and of adherent cells by dead cells) was ignored in all experiments.

After the second transfection with siRNA on day 0, cells were grown in the presence or absence of doxycycline and RSL1 for 4 days, at which time viable (adherent) and dead (nonadherent) cells were determined. The number of dead cells was measured by counting several thousand nonadherent cells in the medium using a Coulter cell counter. The number of viable cells was counted in the same way after detachment of adherent cells from the dish by trypsin treatment. The percentage of dead cells was calculated as the number of nonadherent cells divided by the total number of adherent plus nonadherent cells. Each assay consists of the results for a single well in a six-well plate, which typically contains 0.5 to 1 million cells at the time of the assay. At least 6 independent assays were carried out for each siRNA knockdown experiment and the results were averaged and standard deviations were determined.

### Contraction Assay

As described previously, the DIT7 cells used in the contraction assay carry an integrated copy of the *HPRT* minigene, whose expression is under control of the Tet-ON promoter [35]. The CAG_95_ repeat located in the intron inactivates the minigene by causing aberrant splicing of the mRNA, rendering the protein nonfunctional. Contraction of the repeat to less than 39 units allows sufficient correct splicing to give normal HPRT function. This selection assay measures contractions of 56 to 95 repeat units. In the text we refer to these events specifically as repeat contractions and generically as repeat instability.

For contraction assays, after the second transfection with siRNA on day 0, DIT7 cells were grown in the presence or absence of doxycycline and RSL1 for 2 days. The cells were then re-fed with fresh medium lacking inducers and allowed to recover for one day. On day 3 cells were plated in HAT selection medium (0.1 mM hypoxanthine, 0.4 µM aminopterin, and 16 µM thymine) supplemented with doxycycline at a cell density of 500,000 cells per 10-cm dish and allowed to form colonies. Contraction frequencies were calculated as the number of HPRT^+^ colonies divided by the number of viable cells; they are the average of at least 6 experiments.

### In Vitro Binding and Western Blotting

To test in vitro binding, we designed the following 4 DNA oligos: 13-4 CGGCGCTGGGCGCGCACCGAG**(CAG)_13_**GATCCTCGAGCTGGTCCCGCAGGC; 13-5 CGGCGCTGGGCGCGCACCGAG**(CTG)_13_**GATCCTCGAGCTGGTCCCGCAGGC; 13-7 CGGCGCTGGGCGCGCACCGAGGATCCTCGAGCTGGTCCCGCAGGC; and 13-6bait GCCTGCGGGACCAGCTCGAGGATCCTGCTCGGTGCGCGCCCAGCGCCG-Bio. Annealing 13-6bait with 13-4, 13-5 or 13-7 at a molar ration of 1∶4 forms double strand DNA fragments that contain a CAG_13_ hairpin, a CTG_13_ hairpin, or no hairpin, respectively. These pairs of DNA oligos were incubated with streptavidin magnetic particles (Roche) at room temperature for 30 min with gentle shaking. The beads were washed twice with washing buffer (10 mM Tris-HCl, 1 mM EDTA, 100 mM NaCl, pH 7.5), twice with PBS containing 1% NP-40, and then resuspended in 400 µL PBS containing 1% NP-40. 600 µL of 10% milk were added and the solution was shaken gently at room temperature for 2 hr. The beads were then washed four times with PBS containing 0.5% NP-40. For binding, the beads were resuspended in 400 µL PBS with 1% NP-40, about 150 µg of whole cell extract was added, and the mixture was gently shaken for 2 hours at room temperature. Beads were washed 4 times by resuspension in PBS containing 0.5% NP-40 followed by centrifugation at 3000 rpm for 1 minute. Proteins bound to the beads were eluted by addition of 60 µL of Western blot loading buffer (50 mM Tris pH 6.8, 100 mM DTT, 2% SDS, 0.1% Bromophenol Blue, 10% Glycerol), followed by brief vortexing, incubated at 95°C for 5 minutes, and then centrifuged at 8,000 rpm for 1 min. The supernatant was carefully removed for Western blot analysis. 10 µL of the supernatant was loaded in each lane of 10% SDS/PAGE gels, and 5 µg of whole cell extract was loaded in an adjacent lane to serve as a reference. After the gels were subjected to electrophoresis, the proteins were transferred to polyvinylidene difluoride membranes and incubated with XPA (Santa Cruz) or actin (Sigma) antibodies. Immunoblots were then visualized using an enhanced ECL kit (GE Healthcare).

### **Statistics**

Statistical analyses of significance were conducted using Student’s *t*-test to compare the means and standard deviations, which were derived from multiple experiments.

## Results

### TC-NER Protects against Convergent Transcription-induced Cell Death

We had speculated previously that the stalling of RNAPII at CAG repeats during convergent transcription triggers the cellular stress response that leads to cell death [35], [36]. Since TC-NER functions to remove the hairpins that stall RNAPII, we expected that decreasing the effectiveness of TC-NER would lead to more persistent RNAPII stalling and exacerbate convergent transcription-induced cell death.

**Figure 2:**
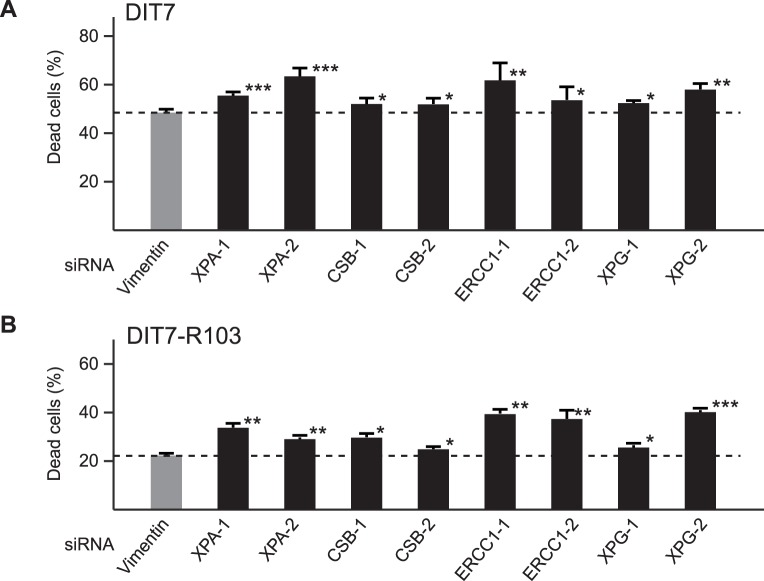
Effects of knockdown of TC-NER components on convergent transcription-induced cell death. (A) siRNA knockdowns in DIT7 cells. Frequencies of cell death are: vimentin, 47%; XPA-1, 55%; XPA-2, 63%; CSB-1, 52%; CSB-2, 51%; ERCC1-1, 61%; ERCC1-2, 53%; XPG-1, 51%; XPG-2, 57%. (B) siRNA knockdowns in DIT7-R103 cells. Frequencies of cell death are: vimentin, 22%; XPA-1, 33%; XPA-2, 31%; CSB-1, 29%; CSB-2, 26%; ERCC1-1, 39%; ERCC1-2, 31%; XPG-1, 25%; XPG-2, 40%. Frequency of cell death was calculated as the number of nonadherent cells divided by the sum of adherent and nonadherent cells. Data are the average frequencies of cell death from at least 6 independent siRNA knockdown experiments. Error bars indicate standard deviations. Statistical significance relative to the vimentin control is indicated: *P<0.05; **P<0.001; ***P<0.0001.

To test the role of TC-NER in convergent transcription-induced cell death, we knocked down four NER components with specific siRNAs and measured the frequency of cell death in DIT7 and DIT7-R103 cells, each of which contains an integrated *HPRT* minigene that carries repeat tracts of CAG_95_ and CAG_15_, respectively (Figure 1). Because DIT7-R103 cells were derived from DIT7 cells by contraction of the CAG repeat, they differ only in the length of the repeat tract [35]. As shown previously, these two cell lines differ in their sensitivity to convergent transcription, with DIT7 cells dying about twice as fast as DIT7-R103 cells when convergent transcription is induced [35]. The NER factors XPA, ERCC1, and XPG, and the TC-NER-specific factor CSB, are required for transcription-induced CAG instability [18], [23]. Treatments with the siRNAs used in this study reduce their target levels by 70% to 90% in human HT1080 cells [18], [23], [32]. siRNA knockdown of XPA, CSB, ERCC1, or XPG significantly increased cell death in both DIT7 and DIT7-R103 cells (Figure 2). These results suggest that TC-NER pathway normally functions to protect cells from convergent transcription-induced cell death, likely by removing the block to the arrested RNAPII complexes, which are the initial triggers for the cell stress response [36].

When the data in Figure 2 are normalized to the vimentin siRNA control for each cell line, it is apparent that knockdown of TC-NER components has a greater effect on cell death in DIT7-R103 (CAG_15_) cells than DIT7 (CAG_95_) cells (Table 1). Thus a cell line with a shorter CAG tract seems to be more sensitive to decreased TC-NER capacity than one with a longer repeat.

**Table 1:** Percentage change in cell death in DIT7 and DIT7-R103 cells due to treatment with specific siRNAs.

siRNA treatment	Increase in cell death (%)^a^	
	DIT7	DIT7-R103
Vimentin [23], [32]	0	0
XPA-1 [23]	17	52
XPA-2 [23]	34	38
CSB-1 [23]	10	30
CSB-2 [23]	8	15
ERCC1-1 [23]	30	68
ERCC1-2 [23]	13	66
XPG-1 [23]	9	16
XPG-2 [23]	22	81
MSH2-1 [23]	−13	−80
MSH2-2 [23]	−11	−55
RNase H1-1 [32]	6	24
RNase H2A-1 [32]	3	25
RNase H1-1+ RNase H2A-1 [32]	15	96

a The percentage increase in cell death due to treatment with a specific siRNA was calculated relative to the vimentin siRNA control as {[(% dead cells after specific siRNA)-(% dead cells after vimentin siRNA)]/(% dead cells after vimentin siRNA)}(100%). The data are from Figures 2, 4, and 5.

### XPA Binds to Hairpins *in vitro*

**Figure 3:**
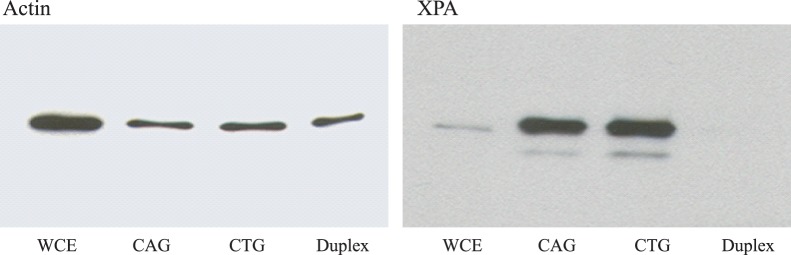
Binding of XPA to CAG- and CTG-containing DNA duplexes. Duplexes without hairpins, with a CAG_13_ hairpin, or a CTG_13_ hairpin were attached to magnetic beads (see Methods) and incubated with a whole cell extract from human cells. The proteins bound to the DNA were then analyzed by Western blot analysis, using antibodies against actin or XPA. Actin served as a control for nonspecific binding. WCE stands for whole cell extract.

Because TC-NER helps to resolve the problems caused by convergent transcription, we sought to determine whether a key component, XPA, might bind to repeat hairpins. XPA is known to bind to helical kinks, which may contribute to the way a cell selects the appropriate DNA repair pathway [37]. In addition, UvrA, a nucleotide excision repair component in E.coli, has been shown to bind to CAG hairpins *in vitro* [38]. To test whether XPA is recruited to the hairpins, we annealed DNA oligos to form a duplex lacking a hairpin, a duplex with a CAG hairpin, or one with a CTG hairpin and then incubated them in a nuclear extract as binding baits. We then performed a pull-down assay using XPA-specific antibody. As shown in Figure 3, XPA binds to CAG and CTG hairpins with similar efficiency, but does not bind to duplex DNA. These results indicate that XPA is likely to be one of the proteins associated with repeat tract hairpins in cells. Because we used a nuclear extract as a source of protein, our results do not distinguish between the binding of XPA directly to the hairpins or via association with other proteins.

### MSH2 Promotes Convergent Transcription-induced Cell Death

**Figure 4:**
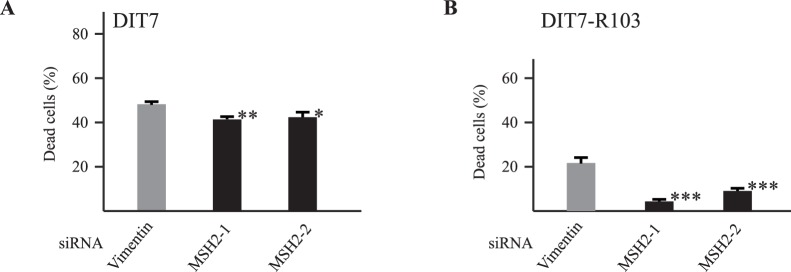
Effects of MSH2 knockdown on convergent transcription-induced cell death. (A) siRNA knockdowns in DIT7 cells. Frequencies of cell death are: vimentin, 47%; MSH2-1, 41%; MSH2-2, 42%. (B) siRNA knockdowns in DIT7-R103 cells. Frequencies of cell death are: vimentin, 22%; MSH2-1, 4%; MSH2-2, 9%. Frequency of cell death was calculated as the number of nonadherent cells divided by the sum of adherent and nonadherent cells. Data are the average frequency of cell death from at least 6 independent siRNA knockdown experiments. Error bars indicate standard deviations. Statistical significance relative to the vimentin control is indicated: *P<0.05; **P<0.001; ***P<0.0001.

The MMR recognition complex MSH2/MSH3 (MutSβ), which binds to CAG and CTG hairpins in vitro [25], [26], is a likely candidate for the cellular component that stabilizes repeat structures to form obstacles for RNAPII [24], [35]. If the stalling of RNAPII is an essential element in the signal for convergent transcription-induced cell death, then we would expect that depletion of MSH2/MSH3 should reduce cell death. To test this idea, we used siRNAs to knock down MSH2 in DIT7 cells and in DIT7-R103 cells. As shown in Figure 4, treatments with two MSH2 siRNAs significantly reduced death in both cell lines. As with the knockdown of TC-NER components, the normalized effect of MSH2 knockdown on cell death was greater in DIT7-R103 (CAG_15_) cells than in DIT7 (CAG_15_) cells (Table 1).

### RNase H Enzymes Reduce Convergent Transcription-induced Cell Death

**Figure 5:**
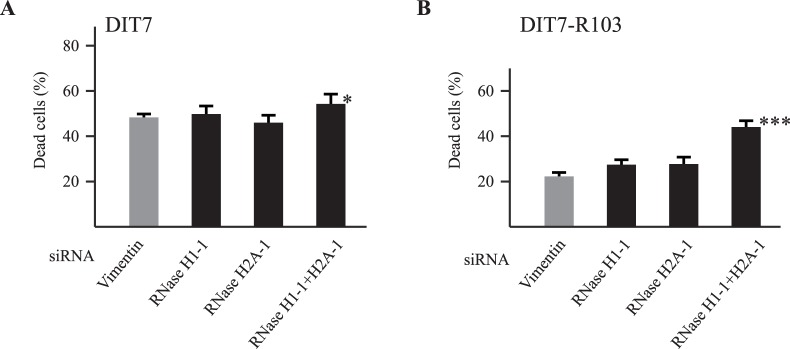
Effects of RNase H knockdown on convergent transcription-induced cell death. (A) siRNA knockdowns in DIT7 cells. Frequencies of cell death are: vimentin, 47%; RNase H1-1, 49%; RNase H2A-1, 46%; RNase H1-1 plus RNase H2A-1, 54%. (B) siRNA knockdowns in DIT7-R103 cells. Frequencies of cell death are: vimentin, 22%; RNase H1-1, 27%; RNase H2A-1, 28%; RNase H1-1 plus RNase H2A-1, 44%. Frequency of cell death was calculated as the number of nonadherent cells divided by the sum of adherent and nonadherent cells. Data are the average frequency of cell death from at least 6 independent siRNA knockdown experiments. Error bars indicate standard deviations. Statistical significance relative to the vimentin control is indicated: *P<0.05; **P<0.001; ***P<0.0001.

We previously showed that extensive RNA/DNA hybrids (R-loops) form during sense transcription of CAG repeat tracts in human cells [32]. RNase H enzymes normally remove the RNA component of R-loops to eliminate the hybrids. Depletion of RNase H1 or RNase H2A, which would prolong the lifetime of R-loops, increases transcription-induced CAG instability in human cells [32], suggesting that R-loops promote repeat instability. We speculated previously that R-loops might enhance hairpin formation in the nontemplate strand [23], [32]. Since hairpins block RNAPII, we expected that depletion of RNase H1 and RNase H2A would increase hairpin formation and RNAPII stalling, and thus increase cell death. To test whether depletion of RNase H enzymes would increase cell death, we used siRNAs to knockdown RNase H1 and RNase H2A. Knockdown of either RNase H1 or RNase 2A alone did not substantially affect cell death in DIT7 cells or in DIT7-R103 cells; however, their double knockdown significantly increased cell death in both cell lines (Figure 5). Once again, the normalized effect of the double knockdown on cell death was greater in DIT7-R103 (CAG_15_) cells than in DIT7 (CAG_95_) cells (Table 1).

### MSH2, XPA, and RNase H Modulate Convergent Transcription-induced Repeat Contraction

**Figure 6:**
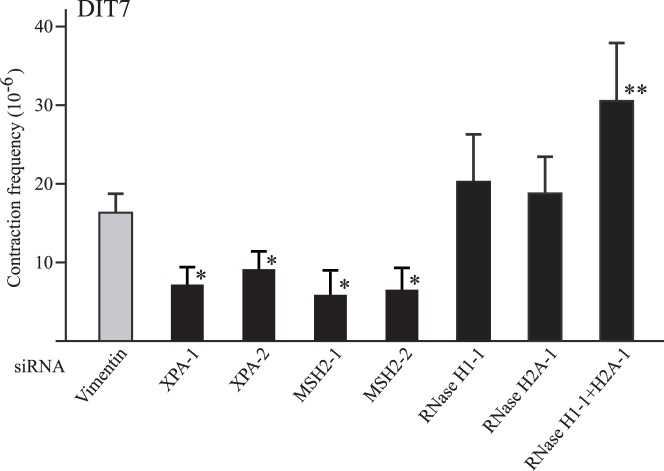
Effects of knockdowns of MSH2, XPA and RNase H on convergent transcription-induced CAG repeat contraction. Contraction frequencies were calculated as the number of HPRT^+^ colonies divided by the number of viable cells, averaged over at least 6 independent siRNA knockdown experiments. Error bars indicate standard deviations. Statistical significance relative to the vimentin control is indicated: *P<0.05; **P<0.01.

In human cells, both TC-NER and mismatch recognition by MSH2/MSH3 are required for repeat contraction induced by sense transcription through the repeat tract, since knockdown of any of the individual components reduces the frequency of transcription-induced CAG repeat contraction [18]. By contrast, RNase H, via its ability to eliminate R-loops, helps to prevent transcription-induced repeat contraction [32]. Since convergent transcription stimulates repeat instability synergistically relative to sense or antisense transcription alone [35], it was unclear whether TC-NER, mismatch recognition, and R-loops would have the same effect on convergent-transcription-induced repeat contraction as they do on instability induced by sense transcription. To test these processes, we measured the CAG contraction frequencies in DIT7 (CAG_95_) cells after knockdown of XPA, MSH2, or RNase H enzymes in cells induced for convergent transcription. As shown in Figure 6, knockdown of XPA or MSH2 significantly reduced contraction frequencies, while simultaneous knockdown of RNase H1 and RNase H2A significantly enhanced the contraction frequency. These results suggest that convergent transcription-induced repeat instability, like that induced by sense transcription alone, also depends on TC-NER and mismatch recognition, and is enhanced by R-loops.

## Discussion

Antisense transcripts are common in human genes [39], suggesting that head-to-head, convergent transcription may be a frequent occurrence on human chromosomes. Antisense transcripts have been found in several trinucleotide repeats (TNR) disease genes, with 8 identified in vivo [17], [40], [41], [42], [43], [44], [45], [46] and at least 10 others in human cell lines [47]. Previously, we examined the biological consequences of convergent transcription through a CAG tract, showing that it promotes repeat instability and causes massive cell death [35]. Here, we have examined the influences of three DNA metabolic processes on convergent transcription-induced cell death and repeat instability. The TC-NER pathway of DNA repair, the mismatch repair recognition component MSH2, and the RNase H species involved in R-loop resolution, which were first identified as playing critical roles in repeat instability induced by sense transcription [18], [23], [32], all affect the repeat instability and cell death induced by convergent transcription. These results suggest that a common structure, generated by convergent transcription through a CAG repeat tract, is likely to be ultimately responsible for both repeat instability and cell death.

For sense transcription-induced repeat instability, we suggested that transcription allowed slipped duplexes to form with looped out CAG and CTG segments [22], [23], and that R-loops enhanced the formation of these aberrant structures [32]. Stabilization of CAG and CTG loops by MSH2/MSH3 (MutSβ) binding can block the progress of RNAPII [24], [25], thereby creating a signal that called TC-NER into play to resolve the block [22], [24], [48]. This working model was created to be consistent with the results from siRNA knockdowns. Depletion of RNase H, which would increase the lifetime of R-loops, would be expected to increase the formation of slipped duplexes, leading to more repeat instability, as observed [32]. Knockdown of MSH2, which would decrease binding to and stabilization of CAG and CTG loops, would reduce stalling of RNAPII, leading to the observed decrease in repeat instability [18]. Knocking down of components of TC-NER prevent the resolution of the block, which is the mechanism by which the repeat is rendered unstable, and thus decrease repeat instability [23]. Here we have shown that this same reasoning applies to convergent transcription-induced repeat instability.

We have speculated elsewhere [35] that convergent transcription through a repeat tract can generate aberrant structures with stalled RNAPII complexes on both strands, creating what we have termed a double bubble [36]. Because the structures on each strand are analogous to the one described above for sense transcription, it was our expectation that knockdown of RNase H, MSH2, and TC-NER would produce the same effects on repeat instability induced by convergent transcription as they do on repeat instability induced by sense transcription. Our results match these expectations: depletion of RNase H increases instability, while depletion of MSH2 and TC-NER decrease repeat instability.

The more surprising result of convergent transcription through a CAG repeat tract–massive cell death–depends on simultaneous induction of both sense and antisense transcription on either side of a CAG repeat tract, so that converging RNAPII complexes encounter the same tract [35]. The resulting double bubble, produced by stalled RNAPII complexes on both strands, must present some significant complication for the cell, which induces an ATR response and triggers cell death, two consequences that are not associated with sense transcription alone [35], [36]. At the outset, it was unclear whether the processes involved in convergent transcription-induced repeat instability would also be involved in the associated cell death. Our knockdown experiments show clearly, however, that RNase H, MSH2, and TC-NER are all involved in both repeat instability and cell death. We can interpret our results in terms of the likely effects on the formation or persistence of the convergent transcription-induced double bubble. Knockdown of RNase H increases R-loops, which favors formation of the slipped duplexes that are key to formation of the double bubbles, thereby increasing the structure formation and increasing cell death. Knockdown of MSH2 prevents stabilization of the CAG and CTG loops, thereby decreasing structure formation and cell death. Similarly, knockdown of MSH3 also reduces cell death, while double knockdown of MSH2 and MSH3 reduces cell death to the same level as either single knockdown (data not shown), consistent with MutSβ playing a role in the stabilization of CAG and CTG loops [25], [26]. Finally, depletion of TC-NER components prevents resolution of the block to RNAPII, prolonging the aberrant structure and increasing cell death.

One striking feature of the effects of siRNA knockdowns on cell death is that DIT7-R103 cells, which carry a short repeat (CAG_15_), are more strongly affected than DIT7 cells, which carry a long repeat (CAG_95_). This counterintuitive result cannot be due to different locations of the repeat in the genome, for example, because DIT7-R103 cells were derived from DIT7 cells by contraction of the repeat. Although we do not know the basis for the difference, we speculate that it reflects the different numbers of CAG and CTG loops that can form in the two repeats. The long CAG tract can potentially form multiple loops, consistent with our measurements of single-stranded regions within the tract [32], while the short tract is unlikely to form more than one. Reduction of MSH2, for example, would reduce the number of stabilized loops in a tract. If the tract has multiple loops, however, some may still be stabilized, resulting in a small effect on cell death. By contrast, in a tract with a single loop, reduction of MSH2 would decrease the number of cells in which the loop is stabilized, thereby reducing cell death. Similar arguments can be made for the effects of knockdowns of RNase H and TC-NER, both of which would be expected to increase the number of stabilized loops. If cells with long repeats already have multiple stabilized loops, an increase may have little effect on cell death, whereas in cells with a single repeat, knockdowns may increase the proportion of cells with a stabilized loop, resulting in more substantial increases in cell death.

Our results are consistent with the idea that the stalled RNAPII is the original signal triggering cell death during convergent transcription [17]. Previous studies showed that agents such as UV light, actinomycin D, psoralen, or antibodies against the RNAPII elongation complex–all of which interfere with transcription by blocking RNAPII genome wide–can stimulate apoptosis [49], [50], [51], [52]. Both genome-wide arrest of RNAPII and its stalling at CAG tracts stimulate a cellular response via the ATR signaling pathway [35], [49]. It is remarkable that RNAPII arrested at a single locus in the genome has such a similar effect on cells as genome-wide transcriptional interference, which occurs at thousands of actively transcribed genes. The critical feature of this locus appears to be the ability of CAG repeats to form abnormal secondary structures capable of blocking transcription on both template strands. It is not yet clear whether convergent transcription-induced cell death is unique to CAG repeats, or is a more general attribute of other structure-forming repeats, as well. Supporting this possibility is the observation that transcription stalls at other types of repeat tracts and at DNA sequences that can form secondary structures in vitro [53], [54], [55], [56], [57]; thus, noncanonical DNA structures can cause problems for RNAP.

The pathogenic mechanisms of CAG diseases are complicated and appear to include toxic proteins and RNA molecules [2], [36], [58], [59]. Convergent transcription-induced cell death raises the possibility that DNA toxicity may also contribute to pathogenesis of these diseases. We showed previously that convergent transcription through CAG repeats can trigger cell death in both proliferating and nonproliferating cells [35], indicating that it is a potential mechanism of cell death in the terminally differentiated cells that are affected in repeat diseases. In addition, antisense transcripts have been found for several TNR disease genes, supporting the idea that convergent transcription occurs in vivo and could potentially affect cell health. The contribution of convergent transcription to the pathogenesis of repeat diseases, however, remains to be tested.

In summary, we have shown that TC-NER pathway, MSH2, and R-loops modulate convergent transcription-induced repeat instability and cell death in human cells. These observations link the mechanisms of convergent transcription-induced repeat instability and convergent transcription-induced cell death, suggesting that a common structure may trigger both outcomes.
